# A new target of radiotherapy combined with immunotherapy: regulatory T cells

**DOI:** 10.3389/fimmu.2023.1330099

**Published:** 2024-01-08

**Authors:** Dongmei Song, Yun Ding

**Affiliations:** Department of Radiation Oncology, The Third Affiliated Hospital of Soochow University, Changzhou, China

**Keywords:** radiotherapy, Tregs, immunotherapy, radiosensitivity, immune microenvironment

## Abstract

Radiotherapy is one important treatment for malignant tumours. It is widely believed today that radiotherapy has not only been used as a local tumour treatment method, but also can induce systemic anti-tumour responses by influencing the tumour microenvironment, but its efficacy is limited by the tumour immunosuppression microenvironment. With the advancement of technology, immunotherapy has entered a golden age of rapid development, gradually occupying a place in clinical tumour treatment. Regulatory T cells (Tregs) widely distributing in the tumour microenvironment play an important role in mediating tumour development. This article analyzes immunotherapy, the interaction between Tregs, tumours and radiotherapy. It briefly introduces immunotherapies targeting Tregs, aiming to provide new strategies for radiotherapy combined with Immunotherapy.

## 1 Introduction

Radiotherapy is a major method in malignant tumour therapy. Studies have shown that about 70% of malignant tumours receive radiotherapy during the course of treatment. On one hand, radiotherapy destroys the DNA structure of tumour cells through direct and indirect effects, leading to cell necrosis and apoptosis (1); On the other hand, it also modifies the tumour microenvironment (TME) that can stimulate and augment the inherent and adaptive immunity of the body in order to achieve a systematic anti-tumour response.

**Figure 1:**
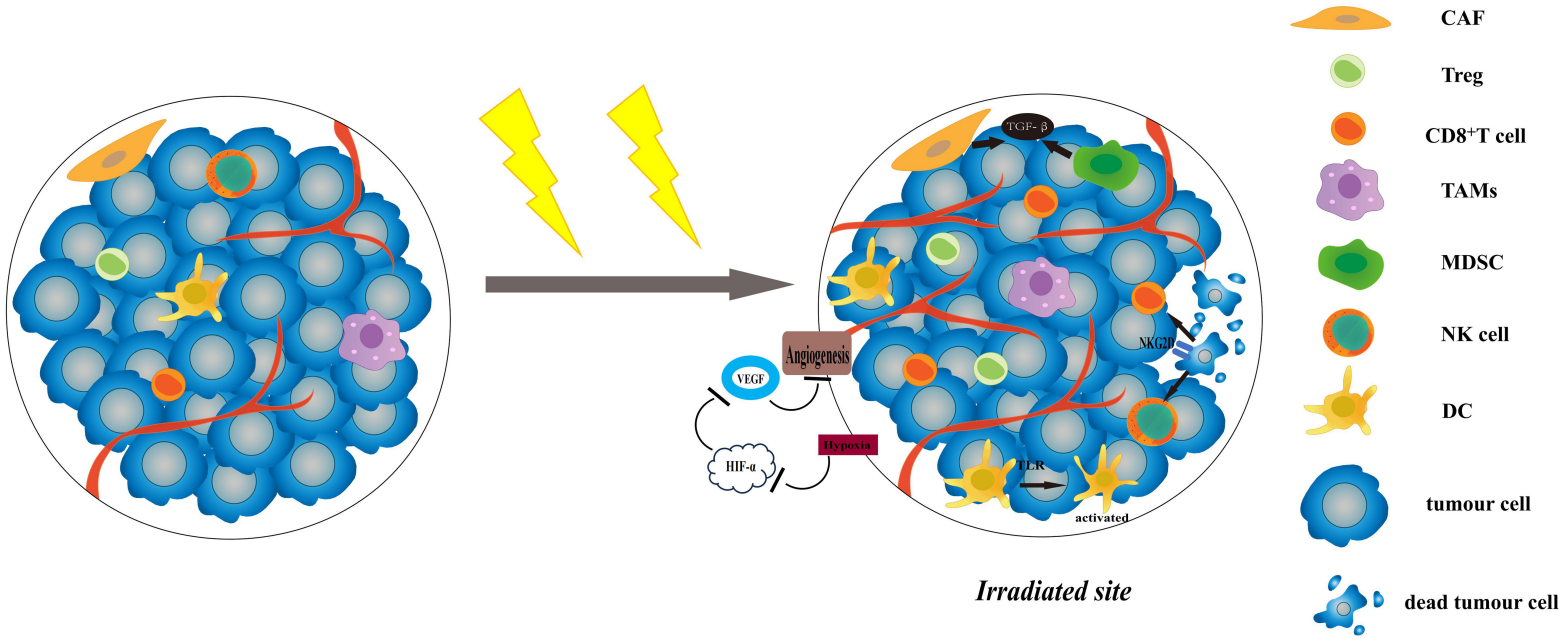
The effects of radiotherapy on TME. Radiotherapy can increase the expression of NKG2D ligands, and promote the killing of NK cells and CD8+ T cells. The increased levels of proinflammatory factors and DAMP-related TLRs can activate DCs and recruit T cells to improve anti-cancer effect. Radiotherapy induces the aggregation of immunosuppressive cells in the TME, like Tregs and MDSCs. It can cause TME hypoxia and induce TGF-β expression on tumour cells to trigger HIF-1/VEGF pathway. CAFs are activated after radiotherapy and can secrete various growth factors, such as TGF-β and MMPs, then further promote tumour growth. TME, tumour microenvironment; NKG2D, NK Group 2 member D; NK cell, natural killer cell; DC, dendritic cell; TRL, Toll-like receptors; MMPs, matrix metalloproteinases; HIF-1, hypoxia-inducible factor 1; VEGF, vascular endothelial growth factor; CAF, cancer-associated fibroblasts.

Radiosensitivity is a key factor affecting the efficacy of radiotherapy. Currently, researches indicate that radiosensitivity is associated with both the biological features and the microenvironment of the tumour (2). The TME is composed of non-cancerous cells, including fibroblasts, blood vessel forming cells, and immune cells (3). These cells are related to tumour growth, invasion and metastasis, and are closely related to the effect of treatment. Complex systemic effects always occur during radiotherapy, it has the potential to produce both anti-tumour and pro-tumour immune response (4). On one hand, most of the antigens expressed by tumour cells are not specific, but tissue differentiation antigens that are also expressed by normal cells (5), and it is difficult to be identified and killed by immune cells. However, when tumour cells death is caused by radiotherapy, they will release tumour-associated antigen(TAAs)and damage-associated pattern molecules (DAMPs), which can activate T cells through antigen presentation (6). At the same time, radiotherapy will induce the production of pro-inflammatory factors, such as type I interferon(IFN) (7), CXCL16 (8) and CXCL10 (9), which recruit T cells into the irradiated TME and kill tumour cells. Radiotherapy can also increase the expression of NK Group 2 member D (NKG2D) ligands on tumour cells and promote the killing of natural killer cells (NK cells) (10). Furthermore, it can also achieve a systematic anti-tumour response through activating the immune system, commonly known as the abscopal effect (11). On the other hand, radiotherapy induces the aggregation of immunosuppressive cells in the TME, such as regulatory T cells (Tregs), tumour-associated macrophages (TAMs) of M2 phenotype, myeloid-derived suppressor cells (MDSCs), and so on (12). These cells hinder the immune response by limiting the activation and proliferation of T cells. It can also cause TME hypoxia and induce TGF-β expression on tumour cells to trigger hypoxia-inducible factor 1(HIF-1)/vascular endothelial growth factor (VEGF) pathway to promote tumour angiogenesis (13, 14). Radiotherapy can induce cancer-associated fibroblasts (CAFs) to secrete various growth factors, such as transforming growth factor β(TGF-β) and matrix metalloproteinases (MMPs), then further promote tumour growth (15) (**Figure 1**).

The efficacy of radiotherapy is limited, using it alone is not enough to destroy the immune tolerance of tumour and kill it all (16). One of the primary problems is that the residual tumour cells are radioresistant and can result in tumour recurrence and metastasis (4). In this case, it is essential to kill the radioresistant tumour cells as many as possible through alternative approaches to optimise tumour control, which has a good development prospect is radiotherapy combined with immunotherapy.

Cancer immunoediting theory states that the process of tumour development can be divided into three stages: Elimination, Equilibration and Escape (17). T cells are an important part of the immune system and can be divided into two categories according to their function in tumour development: CD4^+^ helper T cells (Th cells) and CD8^+^ T cells, which play an anti-tumour role, and Treg cells, which play a pro-tumour role. During tumour development, dendritic cells (DCs) present MHC-I molecules of tumour antigens to CD8^+^ T cells, activating them into cytotoxic T lymphocytes (CTLs) (18), whereas Th cells further promote the proliferation and activation of CD8^+^ T cells (19). CTL can inhibit tumour growth by directly killing tumour cells through secreting granzyme and perforin, or by inducing apoptosis through the Fas/FasL pathway (18). As a type of immunosuppressive cells, Treg cell is an important factor in creating an immunosuppressive TME and promoting tumour growth. Recent studies have demonstrated that decreasing Tregs infiltration can enhance the efficacy of radiotherapy (20, 21). This article focuses on Tregs to introduce the development of radiotherapy combined with immunotherapy in current tumour therapy.

## 2 Immunotherapy

### 2.1 Introduction of immunotherapy

Tumours are genomic diseases, the antigens they expressed are usually the products of mutated cellular genes, aberrantly expressed normal genes, or genes encoding viral proteins (17). These antigens can be recognised by immune system, inducing an immune response that regulates tumour progression. Tumour immunotherapy refers to the method of breaking tumour immune tolerance and inhibiting tumour growth by activating immune cells and enhancing the anti-tumour immune response. It mainly includes: (i) cancer vaccines, such as sipuleucel-T using for prostate cancer (22); (ii) cytokine therapies, such as INF (23), interleukin-2 (IL-2) (24); (iii) Oncolytic virus therapies, such as T-VEC (25); and (iv) chimeric antigen receptor T-cell (CAR-T) and adoptive cell transfer (ACT) (26); (v) antibody therapies or immune checkpoint inhibitors (ICI) such as rituximab and Trastuzumab, as well as CTLA-4 and PD-1 (27, 28). Immunotherapy is a landmark breakthrough in the treatment of tumours and is gradually becoming another pillar of tumour treatment in addition to surgery, chemotherapy and radiotherapy.

### 2.2 Immunotherapy and radiotherapy

Radiotherapy is used to kill tumours through rays. It has a better local tumour control, not only can eliminate tumours and prevent tumour recurrence after surgery, but also can relieve symptoms and improve the quality of life of patients with advanced tumours (11). However, the specificity of radiotherapy is low, while killing the tumour cells, it also damages normal cells and may cause irreversible damage when it exceeds the tolerance of the tissues (29). Most tumours have low sensitivity to radiotherapy, making it difficult to achieve radical tumour cure. On the contrary, immunotherapy has high specificity, most of them can precisely kill tumour cells with fewer side effects. Tumours are usually classified as immunogenic (hot) tumours or non-immunogenic (cold) tumours based on the abundance or paucity of T-lymphocyte infiltration (30). The former has a better efficacy using immunotherapy. Unfortunately, because the majority of tumours are cold tumours, only about 20-40% of patients can benefit from monoimmunotherapy (31).

In order to identify which patients will benefit from immunotherapy, we need to evaluate TME. Considering the dynamic and complex nature of tumour immune regulation, it is difficult to use a single immune biomarker to select patients (32). We usually use assessment methods like immunohistochemistry, flow cytometry, NGS sequencing and others (33). Currently, single-cell protein analysis and single-cell RNA sequencing (scRNA-seq) have been used to assess TME (28), such as melanoma (34), lung cancer (35), renal cell carcinoma (36) and colorectal cancer (37).

One of the major challenges of immunotherapy is how to convert cold tumours into hot tumours to improve efficacy. Radiotherapy has the potential. The main mechanisms responsible for the lack of tumour T-cell infiltration, include tumour antigen deficiency, defective antigen presentation, poor T-cell activation and defective homing to the tumour (38). Radiotherapy can lead to immunogenic cell death that release TAAs (6), and induce increased expression of MHC-I molecules in tumour cells and increase their sensitivity to CTL killing (39), as well as activate the DNA-cGAS-STING pathway to enhance anti-tumour T-cell responses by DCs (7). It also can induce chemokine secretion to recruit T-cells in tumour (11), and at low doses, radiotherapy can normalise dysfunctional tumour blood vessels thus allowing antigen-specific T cells to infiltrate into the tumour tissue (40). Neoadjuvant local low-dose radiation induces iNOS expression in TAMs, triggering efficient recruitment of tumour-specific T cells, which prolongs survival in immune-refractory mouse tumour models (41). Preclinical trials have demonstrated that radiotherapy can convert tumours unresponsive to CTLA-4 into responsive tumours (42, 43).

In summary, radiotherapy and immunotherapy can be mutually reinforcing. There are many existing clinical trials that combine radiotherapy with immunotherapy aiming to improve efficacy (**Table 1**).

**Table 1:** Combined radiotherapy and immunotherapy clinical trials.

Targets	Drugs	Tumour type	NCT number
IL-2	IL-2+RT	metastatic melanoma	NCT01416831
	NHS- IL-2+RT	metastatic non-small cell lung cancer	NCT00879866
CTLA-4	Ipilimumab+RT	metastatic melanoma	NCT01557114
	Ipilimumab+RT	metastatic melanoma	NCT01497808
	Ipilimumab+RT	metastatic disease	NCT02239900
EGFR+CTLA-4	cetuximab+Ipilimumab+RT	locally advanced head and neck squamous carcinoma	NCT02777385
PD-1/PD-L1	Avelumab+RT	Hepatocellular carcinoma	NCT03817736
	Pembrolizumab+SBRT	metastatic non-small-cell lung cancer	NCT02492568
	Pembrolizumab+SBRT	metastatic non-small-cell lung cancer	NCT02444741
	Pembrolizumab+SABR	Oligometastatic renal cell carcinoma	NCT02855203
	Sintilimab+RT	MSI-H/dMMR rectal cancer	NCT04636008.
	Pembrolizumab+SBRT	advanced stage non-small cell lung cancer	NCT04929041
	Durvalumab+RT	non-small-cell lung cancer	NCT04245514
	Toripalimab+IMRT	recurrent nasopharyngeal carcinoma	NCT03854838
CTLA-4+PD-1/PD-L1	Ipilimumab+ Nivolumab+RT	MSS colorectal cancer and pancreatic ductal adenocarcinoma	NCT03104439
	Ipilimumab+ Nivolumab+RT	non-small-cell lung cancer	NCT03217071

## 3 Tregs and tumour

### 3.1 Introduction to Tregs

Regulatory T cells (Tregs) can regulate the response of the immune system to self-antigens, graft antigens, and tumour antigens. Normally, Tregs account for an average of 6% of the peripheral CD4^+^ T cells (44). There are two types of Tregs: natural regulatory T cells (nTreg), which are generated through thymic selection; and inducible regulatory T cells (iTreg), which are transforming from CD4^+^CD25^-^T cells in peripheral blood through microenvironmental induction (45). Except CD25, Tregs also express the cytotoxic T-lymphocyte-associated protein 4 (CTLA-4), the glucocorticoid inducible tumour necrosis factor receptor (GITR), lymphocyte activation gene 3(LAG-3), neuropilin-1, membrane-bound TGF-β, CD62L^high^, CD45RB^low^, CD103, CD5, CD27, CD38, CD39,CD69, CD73, CD122, OX-40 (CD134), CCR4, CCR7, CCR8,TNF-R2 and FOXP3 (46). FOXP3 is an important differentiation marker (47), and the expression of FOXP3 can transform naive CD4^+^ T cells into Tregs (48). The expression of FOXP3 plays a variety of roles, for example, FOXP3 can inhibit the transcription of IL-2 by Treg and promote the expression of CD25 (49). It also reprogrammed the metabolism of Treg, making it play a role in the environment of a low-glucose and high-lactate, and more suitable for the TME (50).

### 3.2 Relationship between Tregs and tumours

Previous studies have shown that Tregs are abundant in the microenvironment of several tumours such as head and neck (51), breast (52), lung (53), liver (54), esophageal (55), gastric (56), pancreatic (57), colon (58) and ovarian cancers (59). There are three possible reasons for its enrichment in the tumour microenvironment: firstly, proliferation: TGF-β secreted by tumour cells can prompt Tregs proliferation in the TME (60); secondly, recruitment: Tregs can be recruited from the peripheral blood to the tumour by interacting with chemokines such as CCL22, which are secreted by tumour cells and macrophages in the TME (59, 61). and thirdly, transformation: TGF-β (62), retinoic acid (63), and others can promote the transformation of CD4^+^Foxp3^-^T cells into Tregs.

Evidences showed that Tregs are associated with tumour prognosis and that high expression of Foxp3 in tumour-infiltrating lymphocytes has been linked to a poor prognosis and a high risk of recurrence (64). Curiel et al. analysed the number of Tregs in the tumour of 70 ovarian cancer patients and found that the increase in Treg counts can led to an increased risk of death and reduced survival in patients (59). Gao et al. found that for patients with hepatocellular carcinoma, the quantity of Tregs in the tumour was inversely related to the overall and disease-free survival of the patients, while positively related to the envelope and vascular invasive capacity of the tumour (65). Bates et al. studied Tregs in tumours of breast cancer patients and found that increased levels of Tregs significantly shortened patients’ overall survival and recurrence-free survival, and the accumulation of Tregs was a marker of breast cancer progression (66). McCoy et al. investigated the relationship between the number of Tregs in tumour tissue and prognosis in rectal cancer patients who had received preoperative chemoradiotherapy. The results showed that patients with fewer Tregs tended to achieve complete pathological remission and had longer survival (67).

Tregs regulate the immune response of cells in the tumour microenvironment through the following mechanisms:(a) secreting immunosuppressive factors, such as TGF-β and IL-10, which enhances immunosuppression by decreasing CD8+ T-cell toxicity, suppressing CD4+ T-cell differentiation, promoting Treg transformation, and inhibiting NK-cell proliferation (68).(b) killing effector T cells and antigen-presenting cells by granzymes and perforins (45). (c) interfering with normal immune cell metabolism and functions, such as depriving IL-2 to interference CD4+ T cells activation (69), hydrolysing ATP to produce adenosine to inhibit effector cell proliferation (70). (d) interacting with DCs to achieve immunosuppression: by binding to DCs through CTLA-4,not only inhibits T cell activation, but also triggers indoleamine 2,3-dioxygenase (IDO) expressed by DCs to suppress T cell proliferation (71), and expands Tregs (72). (e) Enhanced immunosuppressive microenvironment through interaction with MDSCs, such as secretion of IL-10 and TGF-β by MDSCs on stimulation with IFN-γ to induce Tregs (73), and secretion of TGF-β and IL-35 by Tregs to enhance the suppressive function of MDSCs (74). Additionally, an *in vitro* study showed that the presence of large numbers of Tregs can disable the effector function of self-antigen and tumour antigen-specific CD8+ T cells (75). These incompetent CD8+ T cells have a naïve phenotype and respond poorly to tumour antigen stimulation, that is to say, they have a low proliferative capacity after antigen stimulation, produce little IL-2 and other cytokines, and have an up-regulated expression of CTLA-4 (76). Tregs inhibit the body’s immune response through the above actions, thereby promoting tumour growth and affecting the efficacy of treatment.

## 4 Radiotherapy and Tregs

### 4.1 Effect of radiotherapy on Tregs

Different immune cells have different radiosensitivity whereby Tregs are more resistant to radiation than other T and B cells (11). Radiation does not enhance the suppressive function of Tregs in the physiological situation. However, in the tumour environment, Tregs acquire a highly suppressive phenotype, termed tumour-infiltrating effector Treg cells, with increased expression of FOXP3 and CD25 (76) and upregulation of a variety of cell-surface molecules including CTLA-4, GITR, OX40, TIGIT, LAG-3, and TIM-3 (77), that is further enhanced by radiotherapy (78). These Tregs have a stronger immunosuppressive function than the Tregs during normal physiology. It was previously stated that the suppressive function of Tregs is significantly dependent on TGF-β and IL-10, and radiation can affect these factors. A previous study found that low-dose irradiation inhibited IL-10 production, whereas high-dose irradiation (HDR) stimulated IL-10 production (79). In contrast, Hussien et al. noted a significant reduction of IL-10 in serum after HDR (80). The reason for the difference may be due to the fact that 2Gy rays were used in the former, whereas 5Gy rays were used in the latter. Both high and low doses of irradiation can consistently activate TGF-β (2). Dong et al. found that HDR may induce TGF-β1 secretion via activating the cAMP-PKA signaling pathway or inhibiting the PLC-PIP2 signaling pathway, and IL-10 secretion was increased (81). The experiments of Jobling et al. demonstrated that after irradiation, reactive oxygen species(ROS) can induce a conformational change in latency-associated peptide (LAP)-TGFβ1, triggering TGF-β1 release (82).

Radiation can also improve Tregs suppression by several mechanisms:(a) Tregs express the nucleotide exoenzymes CD39 and CD73, which catabolise nucleotides to produce adenosine for immunosuppression, and radiation can enhance the expression of CD39 (83). (b) Apoptotic tumour cells can induce the production of tolerogenic DCs, which increase their inhibitory capacity by enhancing their interaction with Tregs (78). (c) Radiation increases the Akt expression in Tregs (84, 85). Akt, through the DNA-PK/AKT/GSK3β pathway, can mediate the overexpression of cyclin D1, activate DNA damage response, and improve Tregs resistance to radiation-induced apoptosis (86). (d) Radiation induces the production of TGF-β in TME, which stimulates Treg aggregation (87). As a result, the number and suppressive capacity of Tregs in the TME are enhanced by radiotherapy, consequently strengthening the immunosuppression of the TME and affecting tumour therapy.

Several clinical trials have shown an increased number of Tregs in their peripheral blood following radiotherapy for patients with head and neck tumours (88), cervical cancer (89), glioma (90), and colorectal cancer (91). Son et al. showed that radiation increased the proportion of Tregs in irradiated tumours of lung and colon cancer (20). Kachikwu et al. found an increase in Treg cells in spleen, lymph nodes, blood and lungs after local irradiation of tumours in mice (92). Baba et al. irradiated mice inoculated with fibrosarcoma, and found that the percentage of Treg cells in the irradiated mice increased, and their function was not weakened. By using annexin V to detect the apoptotic ability of Tregs, it was found that their apoptotic ability decreased after radiation, indicating that they have a good anti-apoptotic ability against radiation (48). Schuler et al. found that Tregs increased and persisted after radiotherapy in patients with head and neck tumours and were highly expressive of CD39, LAP, GARP and anti-apoptotic proteins. These Tregs are resistant to activation-induced cell death and may lead to cisplatin resistance and tumour recurrence and metastasis (88). Qinfeng et al. conducted tumour biopsies on 59 cervical cancer patients before and after radiotherapy. The study demonstrated that radiotherapy induced the redistribution of immune cells in the tumour environment, specifically manifested as: CD8+T and CD4+T cells decreased, while Tregs did not change significantly, resulting in an impaired anti-tumour immune response (89). According to Balogh et al, consistently high absolute numbers of Tregs could be detected in the spleen of mice irradiated with 2 Gy or higher doses at different time points, and radiation promoted Tregs proliferation and enhanced CTLA-4 and IL-10 mRNA expression (93).

### 4.2 Effect of Tregs on radiotherapy

How to overcome radiation resistance to improve radiosensitivity is the key to improve the effect of radiotherapy. Previous studies have revealed that various pathways can be used to increase Tregs depletion, reduce their function, and improve radiosensitivity. The following are some related studies (**Table 2**).

**Table 2:** The targets for Tregs.

Molecule(s)/drug	Function of the molecule(s)/drug
IL-2/CD25	constitutively express high levels of the high-affinity IL-2 receptor consisting of CD25, CD122 and CD132, Tregs can restrict T cell activation by “robbing” exogenous IL-2
CTLA-4	Tregs can compete with conventional T cells to bind CD80/CD86 through CTLA-4 and prevent the activation of effector T cells
GITR	constitutively expressed in CD4+ FoxP3+ Tregs, activation of the GITR signaling pathway leads to instability and depletion of Tregs, it also acts as a co-stimulatory factor on effector T cells and stimulates T cell proliferation and activation
Chemokines and their receptors	expressed in Tregs, can mediate the migration of Tregs to the TME
Cyclophosphamide	interferes with DNA replication, it can selectively reduce Tregs at low dose
PD-1/PD-L1	PD-L1 can cooperate with TGF-β to promote iTreg transformation, and enhance its function by maintaining and enhancing the expression of Foxp3
TGF-β	inhibit anti-tumour immunity by activating SMADs and NFAT to induce the expression of FOXP3 in CD4+ T cells, promote their differentiation to a Treg phenotype
Others	STAT3 can enhance Tregs function and increase their conversion; the expression of FOXP3 can transform naive CD4+ T cells into Tregs;5-FU can reduce Tregs without affecting CD4+ T cells

#### 4.2.1 IL-2 and CD25

It has been proposed that IL-2 plays a “key role” in the activation of Tregs and is required for the sustained expression of Foxp3 and CD25 in natural Tregs, as well as the improvement of their function (94, 95). A main feature of Tregs is that they constitutively express high levels of the high-affinity IL-2 receptor consisting of CD25, CD122 and CD132, but produce little IL-2 themselves. This means that Tregs can restrict T cell activation by “robbing” IL-2 (96). The addition of exogenous IL-2 can eliminate Tregs suppression *in vitro* (97). Thus, IL-2 and CD25 may be targets for controlling Tregs survival and suppressive function (98), and the combination of radiotherapy with this approach may enhance anti-tumour effect. After radiotherapy, there are an increase in CD25, and a decrease in CD122 (99). Yasuda et al. ‘s study in rectal cancer found that mice treated with RT+IL-2 had a higher percentage of CD4^+^T cells but a lower percentage of Tregs and MDSC in splenocytes, Administering IL-2 significantly improved the anti-tumour impact of RT (100). Oweida et al. developed a mouse head and neck tumour model using anti-CD25 antibodies to neutralise Tregs activity. They found that compared with radiotherapy alone, mice treated with combination therapy had more effector T cell infiltration in their tumours. And the combination therapy promoted more macrophages conversion to the M1 phenotype, resulting in more tumour regression (21). Kachikwu et al. also used anti-CD25 antibodies to deplete Tregs, and in prostate cancer, when combined with radiotherapy, tumour growth was delayed and temporary shrink, with improved efficacy compared to radiotherapy alone (92). Piper et al. used PD1-IL2v, a variant of IL-2 immunocytokine that abolishes the binding with CD25 and targets PD-1. It can enhance the infiltration and activation of tumour antigen-specific T cells and reduce Treg cells. Combining it with radiotherapy can significantly improve the efficacy of radiotherapy (99). A phase II clinical trial has shown that in metastatic melanoma, patients treated with IL-2+SBRT have a higher disease control rate compared with IL-2 monotherapy. 44 patients were included in the research. The result showed that the ORR in the SBRT + IL-2 group was 54%: 21% complete response (CR), 33% partial response (PR), 21% stable disease (SD) and 25% progressive disease (PD). The ORR in patients receiving IL-2 monotherapy was 35%: 15% CR, 20% PR, 25% SD and 40% PD (101).

#### 4.2.2 CTLA-4

APC activates T cells by presenting antigens, with MHC as the primary stimulatory signal and CD80/86 as the secondary co-stimulatory signal interacting with TCRs and CD28 on T cells, respectively (102). CTLA-4 expressed by Tregs and CD28 expressed by conventional T cells (Tconv) have common ligands CD80 and CD86, but the affinity of CTLA-4 is significantly higher than CD28, so Tregs can compete with conventional T cells to bind CD80/CD86 and prevent the activation of effector T cells (103). In addition, CTLA-4 on Tregs can downregulate the expression of CD80 and CD86 on DCs, further impeding the activation of conventional T cells (104, 105). It has been demonstrated that CTLA-4 blocker enhances anti-tumour response mainly by selectively reducing Tregs, with T cell activation being a secondary role (106). Therefore, when RT combining with CTLA-4 blockage therapy, more durable T-cell activation can be achieved and the efficacy of radiotherapy can be increased.

In a study of poorly immunogenic metastatic breast cancer 4T1, Demaria et al. combined CTLA-4 blocker with radiotherapy and found a significant reduction in tumour growth rate. Efficient CD8^+^ T cell-dependent anti-tumour immunity was formed, which not only controlled local tumour growth but also inhibited the formation of lung metastasis (42). Ji et al. found that radiotherapy combining with CTLA-4 blocker reduced the percentage of Tregs and delayed tumour growth and metastasis compared to radiotherapy alone (107). Ipilimumab is a human monoclonal antibody against the CTLA-4 antigen, and several studies have reported significant effects of combination of radiotherapy and Ipilimumab in the treatment of metastatic melanoma (108), non-small cell lung cancer (109) and prostate cancer (110).

#### 4.2.3 GITR

GITR is a co-stimulatory molecule that is expressed at low levels in resting CD4^+^ and CD8^+^ T cells and is constitutively expressed in CD4^+^ FoxP3^+^ Tregs (111). Activation of the GITR signaling pathway leads to instability and depletion of Tregs (112). Furthermore, GITR signaling acts as a co-stimulatory factor on effector T cells and stimulates T cell proliferation and activation (113). Schoenhals et al. showed that anti-GITR antibody could reverse the increase in Tregs induced by radiotherapy and increase the efficacy of radiotherapy. They also compared the efficacy of G1 and G2 antibodies, in which the G1-type antibody only agonises the GITR, whereas the G2-type antibody agonises the GITR signaling pathway and depletes Tregs meanwhile. The mice receiving the G2 antibody treatment had a more significant tumour regression and experienced longer survival, the abscopal effect was also observed, indicating that depletion of Tregs can increase the efficacy of radiotherapy (114).

#### 4.2.4 Chemokines and their receptors

Tumour-infiltrating macrophages and tumour cells can produce chemokine ligand 22 (CCL22), which attracts cells expressing CCR4 to the tumour tissue (115). CCR4 is primarily expressed in effector Tregs, rather than naïve Tregs and Th2 cells. A study by Sugiyama et al. suggested that anti-CCR4 antibodies can selectively deplete effector Treg cells and increases tumour antigen-specific CD4^+^ and CD8^+^ T cells (116); an *in vitro* study using the antibody to neutralise CCL22 blocked Tregs migration to tumour tissue (59). These trials inspired us that blocking the transport of Tregs is also a potential therapeutic strategy. Similarly, CCR6, a chemokine receptor selectively expressed in Th17 cells and Tregs, can mediate the migration of circulating Tregs to the TME through the CCL20-CCR6 axis, leading to tumour progression and poor prognosis (117). Rutihinda et al. discovered that radiation promotes the secretion of CCL20 by tumour cells and inhibition of the CCL20-CCR6 axis reduces the infiltration of Tregs into the TME, thus inhibiting tumour growth and improving tumour response to radiation (118).

#### 4.2.5 Cyclophosphamide

Cyclophosphamide is an alkylating agent that interferes with DNA replication and kills highly proliferating cells. High dose administration of cyclophosphamide has severe effects on all T cells. However, when used at low dose for long time, it has been shown to selectively reduce Tregs, resulting in enhanced anti-tumour immune responses in both humans and rodents (119–121). Zhao et al. attribute the ability of low-dose CTX to target and deplete Tregs to the down-regulation of a miRNA (miR-142-3p) and up-regulation of the nucleotide exonuclease CD39, which made the ATP levels lower than conventional T cells, increasing the sensitivity of Tregs to CTX (122). North et al. showed that cyclophosphamide eliminated CD4^+^ T cells that induced CD8+ T cell suppression and promoted immunotherapy of tumours (123). Son et al. demonstrated that radiotherapy combined with low-dose CTX significantly could reduce the number of Tregs in rectal and lung cancers while increasing the number of effector T-cells, tumours regression and survival rate in mice have been improved. Compared with anti-CD25 antibody, LD-CTX combined radiotherapy more effectively inhibited the growth of primary and distant tumours and significantly improved the survival rate of mice (20).

#### 4.2.6 PD-1/PD-L1

In many tumours, immune cells are highly expressive of PD-1 (47). When CD4^+^ effector T cells in the TME express PD-1, they are actually transformed into Tregs (124). PD-L1 can not only cooperate with TGF-β to promote iTreg transformation, but also enhance its function by maintaining and enhancing the expression of Foxp3 (125). Increased PD-L1 expression in tumour cells after X-ray irradiation aggravates the immunosuppressive nature of the TME (3). Sharabi et al. used radiotherapy combined with PD-1 blockers to treat melanoma mice and found that that the combined therapy reversed the increase of Tregs, increased the ratio of CD8^+^ T cells to Tregs and improve prognosis compared with radiotherapy alone (126). Since anti-CTLA-4 and anti-PD-L1 use non-redundant mechanisms and act at different time points in the anti-tumour immune response, dual immune checkpoint inhibition in combination with RT may be a future therapeutic option (127). Ji et al. used a combination of radiotherapy, anti-CTLA-4, and anti-PD-L1 blocker to treat mice inoculated with colorectal cancer cells. When compared to using radiotherapy alone or combining it with anti-CTLA-4, the combined treatment led to a more significant decrease in Tregs, an increase in CD8^+^ T cells, and led to a more effective therapy (107).

#### 4.2.7 TGF-β

TGF-β is an important factor in the regulation of immune homeostasis and immune tolerance, and it regulates the generation and functions of many kinds of immune cells (128). In TME, TGF-β is mainly produced by tumour cells and several other types of cells, including Tregs, fibroblasts, macrophages, etc (129). Elevated levels of TGF-β can inhibit anti-tumour immunity by activating SMADs and NFAT to induce the expression of FOXP3 in CD4^+^ T cells, which can promote their differentiation to a Treg phenotype (130). As previously stated, irradiation can continuously activate TGF-β (2), the activated TGF-β reduces cellular radiosensitivity by decreasing DNA damage and apoptosis (131). It has been shown that inhibiting TGF-β signaling reduces the number of Tregs and restores the tumour sensitivity to anti-PD-1 and anti-CTLA-4 therapy (132), and blocking TGF-β prior to radiotherapy increases clonogenic cell death and decreases tumour growth (133). A study constructed by Vanpouille-Box et al. found that PD-1-mediated immune escape limits the effect of TGF- β blockers combined with RT, while combination with anti-PD-1 therapy could further improve the efficacy (134). Rodriguez-Ruiz et al. demonstrated TGF-β blockade in conjunction with the anti-PD-1 plus anti-CD137 mAb combination can enhance the abscopal effects of radiotherapy (135). Currently, Yi et al. have developed anti-TGF-β/PD-L1 bispecific antibodies (YM101 and BiTP), which activities were superior to anti-TGF-β and anti-PD-L1 monotherapy, they could effectively inhibit TGF-β1-induced Treg differentiation and suppress the proliferation of tumour cells to slow down tumour growth (136, 137). However, their role with radiotherapy needs further study.

#### 4.2.8 Others

STAT3 can enhance Tregs function and increase their conversion. Oweida et al. used STAT3 antisense nucleotide (ASO) to deplete Tregs, when combined with radiotherapy increased the efficacy of radiotherapy for head and neck tumours (21). Mondini et al. used FOXP3-DRT mice and normal mice to establish a head and neck tumour model, and depletion of Tregs using DT targeting resulted in a significant reduction in tumour burden compared to radiotherapy alone (138). 5-fluorouracil (5-FU) is often used in patients with locally advanced rectal cancer during neoadjuvant chemoradiotherapy and has been shown to induce immunogenic tumour cells death (139). Maeda et al. analysed blood samples from 27 colorectal cancer patients treated with 5-FU-based chemotherapy regimens, and found that there was a significant reduction in Tregs after chemotherapy without affecting CD4^+^ T cells (140). It was a selective Tregs depletion that made 5-FU a radiosensitizer.

## 5 Prospect

Radiotherapy not only kills tumour cells directly, but also regulates the immune response of tumours by reshaping the immune microenvironment, making it possible for radiotherapy combined with immunotherapy to achieve a “1 + 1>2” effect. Nowadays, immunotherapy is developing rapidly, more and more tumours can be treated with radiotherapy combined with immunotherapy to achieve a good tumour control. As a kind of immunosuppressive cells, Treg cells participate in the formation of self-immune tolerance, but also hinder the immune system’s surveillance of tumour cells, promoting tumour progression. Therefore, based on the depletion of Treg cells, immunotherapy combined with radiotherapy has a good development prospect. Nowadays, most combination therapies only exist in animal experiments and still require preclinical and clinical experimental data to confirm. However, enhancing anti-tumour immunity and enhancing the efficacy of radiotherapy by affecting Tregs has great development prospects and is worth our in-depth research.

## Author contributions

DS: Writing – original draft. YD: Writing – review & editing.
